# Analysis of protein composition of rabbit aqueous humor following two different cataract surgery incision procedures using 2-DE and LC-MS/MS

**DOI:** 10.1186/1477-5956-9-8

**Published:** 2011-02-09

**Authors:** Miroslava Stastna, Ashley Behrens, Peter J McDonnell, Jennifer E Van Eyk

**Affiliations:** 1 Johns Hopkins Bayview Proteomics Center, Department of Medicine, Division of Cardiology, Johns Hopkins University, Baltimore, MD, USA; 2 The Wilmer Ophthalmological Institute, Johns Hopkins University, Baltimore, MD, USA; 3 Institute of Analytical Chemistry of the ASCR, v.v.i., Brno, Czech Republic

## Abstract

**Background:**

The aqueous humor (AH), a liquid of the anterior and posterior chamber of the eye, comprises many proteins with various roles and important biological functions. Many of these proteins have not been identified yet and their functions in AH are still unknown. Recently, our laboratory published the protein database of AH obtained from healthy rabbits which expanded known protein identifications by 65%. Our present study extends our previous work and analyses AH following two types of cataract surgery incision procedures (clear corneal and limbal incisions) by using two dimensional gel electrophoresis (2-DE) and liquid chromatography tandem mass spectrometry (LC-MS/MS). Although both incision protocols are commonly used during cataract surgeries, the difference in protein composition and their release into AH following each surgery has never been systematically compared and remains unclear. The first step, which is the focus of this work, is to assess the scale of the protein change, at which time does maximum release occurs and when possible, to identify protein changes.

**Results:**

Samples of AH obtained prior to surgery and at different time points (0.5, 2, 12, 24 and 48 hours) following surgery (n = 3/protocol) underwent protein concentration determination, 2-DE and LC-MS/MS. There was a large (9.7 to 31.2 mg/mL) and rapid (~0.5 hour) influx of proteins into AH following either incision with a return to baseline quantities after 12 hours and 24 hours for clear corneal and limbal incision, respectively. We identified 80 non-redundant proteins, and compared to our previous study on healthy AH, 67.5% of proteins were found to be surgery-specific. In addition, 51% of those proteins have been found either in clear corneal (20%) or limbal incision (31%) samples.

**Conclusions:**

Our results imply that a mechanism of protein release into AH after surgery is a global response to the surgery rather than increase in amount of protective proteins found in healthy AH and a mechanism of protein release for each type of incision procedure could be different. Although the total protein concentration was increased (at 0.5 and 2 hour time points and between types of surgery) many of 2-DE protein spots were similar based on 2-DE and MS analyses, and only a small number of protein spots changed with either the time points or surgical conditions (0.4 -1.9%). This suggests that the high protein content is due to an increase in the concentration of the same proteins with only a few unique proteins being altered per time point and with the different surgery type. This is the first report on the comparison of AH protein composition following two different cataract surgery procedures and it establishes the basis for better understanding of protein release into AH during events such as cataract surgery or other possible intervention to the eyes.

## Background

Cataract surgery is a common surgical procedure for treatment of a cataract, an opacity or cloudiness of the normally clear lens of the eye. In cataract surgery, the clouded lens is removed and replaced by a clear artificial implant through the eye incision into the empty lens capsule. In the limbal procedure, an approximately 11 mm incision is created to extract the lens and closed with multiple sutures. More recently, smaller instruments and foldable lens implants [1-4], have allowed the surgery to be performed with a microincisional procedure. The clear corneal procedure [5] requires a 3 mm incision and uses a phacoemulsification device to break the lens into small fragments by means of ultrasound energy with the fragments aspirated from the eye and a foldable lens implant is inserted through a self-sealing small incision. Compared to the classical limbal procedure, clear corneal incision exhibits less postoperative inflammation, considerably decreases the time required to perform the surgery and reduces time for vision recovery. On the other hand, there are several concerns regarding the use of the clear corneal procedure - the risk of wound leakage and inflow of extraocular fluid in unsutured corneal incision with possible postoperative ingress of bacteria into the anterior chamber [6,7] and a failure in the mechanism of bacterial clearance from the anterior chamber related to less postoperative inflammation [8], which otherwise will prevent or decrease a risk of a clinical disease.

We and others have hypothesized that during and after cataract surgery, various proteins are released into AH as a response to the eye intervention, and the protein composition differs based on distinct surgery procedures performed. Although papers have been published on protein analyses of AH, they were mostly targeted to several specific proteins or the protein detection methods used were other from present-day high sensitive mass spectrometry (MS) [9-13]. Only several papers have been found to contain the larger sets of proteins identified by MS [14-18], mostly from human AH. Previously, we published the protein database of AH in healthy New Zealand white rabbits comprising 98 non-redundant proteins by using extensive separation/MS strategy [19]. In that study, many proteins within the aqueous humor were found to be involved in protection mechanism and were grouped into several functional families: cell adhesion and wound healing, proteases and protease inhibitors, anti-oxidant protection and antibacterial and anti-inflammatory proteins. The literature on previous AH protein analysis as well as on the specific proteins identified is discussed in more details in aforementioned paper [19].

In present study, the AH protein composition changes in response to different severity of injury are presented in a timeframe of 48 hours following the surgery and specific proteins are detected and discussed. Two incision procedures commonly used during cataract surgery were performed in a rabbit model, clear corneal and limbal incisions. Rabbit experimental model was used for several reasons. First, rabbit is one of the common ophthalmic animal models which is often used. Certainly, the rabbit represents the standard model for e.g. glaucoma surgery. Second, rabbit eyes are closer in size and structure to human than rodent eyes which means techniques developed in the rabbit can be more readily adapted to humans. Third, the volume of AH obtained from rabbit eye is greater (around 0.2 mL) than either mouse or rat which means we could obtain sufficient amount of sample for proteome analysis. Samples of AH were taken at 5 time points (0.5, 2, 12, 24 and 48 hours) after each surgery type, each time point including three different animals, and subjected to 2-DE. The patterns of silver stained protein spots in 2-DE gels were evaluated by gel image analysis software and proteins in spots were identified by LC-MS/MS.

## Methods

### AH collection

The adult (males) New Zealand white rabbits (2.5-3.0 kg) were provided by an authorized breeding center and were kept in individual cages under well-defined and standardized conditions in humidity and temperature controlled room. Before surgery, rabbits were anesthetized with intramuscular ketamine (Ketaject, Phoenix Pharmaceutical Inc., St. Joseph, MO; 45 mg/kg of body weight) and xylazine (Xyla-Ject Phoenix Pharmaceutical Inc., St. Joseph, MO; 4.5 mg/kg of body weight), the eyes were treated with 5% povidine iodine and after appropriate draping, a lid speculum was inserted in the eye to keep it open. After topical anesthesia by proparacaine 0.5% eyedrops (Alcaine^®^, Alcon, Ft. Worth, TX), two types of incisions used as part of cataract surgeries were performed either a self-sealing clear corneal incision of 3 mm in width with no sutures or a limbal incision 11 mm in width closed with five nylon 10-0 sutures after surgery. The samples of AH were collected at five time points (three animals for each time point) for both types of incisions (0.5, 2, 12, 24 and 48 hours) through paracentesis under direct microscopic visualization (Zeiss S8, Carl Zeiss, Thornwood, NY). The samples of AH without surgery (pre-surgery) were collected from five healthy rabbits via paracentesis as well. Paracentesis was performed through the center of the cornea using a 27-gauge needle attached to a 1-ml tuberculin syringe. The central location of the paracentesis was selected to avoid contact with other intraocular structures such as the iris and the anterior lens capsule and the possible subsequent release of non-involved proteins. Similarly, the needle bevel was directed downwards at the moment of insertion, to allow for minimal excursion of the tip into the anterior chamber. AH samples of approximately 0.2 ml were collected from one eye of each rabbit and frozen at -80°C immediately.

All experiments were conducted in accordance with the Principles of laboratory animal care (NIH publication No. 85-23, revised 1985), Institutional Animal Care and Use Committee from Johns Hopkins University, the OPRR Public Health Service Policy on the Humane Care and Use of Laboratory Animals (revised 1986), and the US Animal Welfare Act, as amended, and were approved by the Swiss Federal and local Ethics and Agricultural Committees.

### Protein concentration measurement

**Figure 1:**
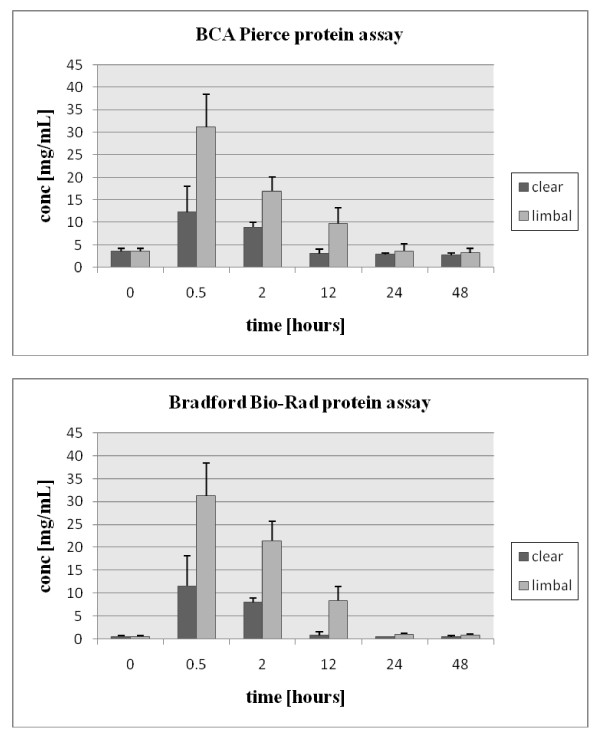
**Protein concentration measurements**. Full description in the text.

Protein concentration of AH samples was determined by both the BCA protein assay (bicinchoninic acid, Pierce, Rockford, IL) and Bio-Rad protein assay (Bradford dye-binding procedure [20], Hercules, CA) with bovine serum albumin as a standard (Figure 1). Each time point in Figure 1 reflects the average concentration value with standard deviations calculated for 5 animals (time point at 0 hour) and for 3 animals (time points at 0.5-48 hours) for each surgical procedure, respectively. The volumes for each of the collected rabbit AH samples ranged from 0.15 to 0.2 mL. Whereas the concentration values for high concentration samples (0.5 and 2 hour time points) are very similar for both protein assays in clear corneal samples (8.9 mg/mL in average according to BCA Pierce protein assay and 8.0 mg/mL according to Bio-Rad Bradford based protein assay at 2 hour time point), the greater concentration differences were observed for low concentration samples (0, 24 and 48 hour time points). For example, protein concentration measured by BCA Pierce protein assay was an average 4.5 times higher in pre-surgery samples and an average 5.5 times higher in clear corneal incision samples (48 hour time point) compared to Bio-Rad Bradford based protein assay reading. The reasons for these discrepancies can be diverse, we assume that one of them is a lower dilution of low concentration samples during protein assays compared to high concentration samples, which can result in higher concentration of components in sample interfering with protein assays. Also, a search of the literature shows that both protein assays have been used in the past for AH protein concentration determination, with higher values obtained by using BCA Pierce protein assay [9,21-24]. Healthy AH protein concentration is reported mostly in range from 0.8 to 2.5 mg/mL for different animals by using BCA protein assay [21,22] and in range from 0.05 to 0.23 mg/mL by using Bradford protein assay [9,23,24]. We used Bio-Rad Bradford based protein assay throughout this study for protein amount calculation.

### 2-DE

2-DE was carried out using Protean IEF Cell (Bio-Rad, CA) for 1^st ^dimension and Protean II xi Cell for 2^nd ^dimension. Polyacrylamide gel strips with an immobilized pH gradient of 4-7 (180 × 3 × 0.5 mm, GE Healthcare Bio-Sciences AB, Uppsala, Sweden, Cat.# 17-1233-01) were used for 1^st ^dimension. As the limited numbers of proteins has been observed outside of pH range 4-7 for AH [19], we used this narrower pH range, which can also improve the resolution of protein spots. AH samples were solubilized in IEF buffer (8 M urea, 2 M thiourea, 4% (w/v) CHAPS, 1% (w/v) DTT, 1% (v/v) HED, 0.2% (v/v) carrier ampholyte (pH 4-7) and 0.005% (w/v) bromophenol blue) and loaded into a focusing tray with the strip placed gel side down with a total volume of 350 μl. The strip was rehydrated at 50 V for 12 hours to enhance protein uptake and subjected to a voltage ramping up to 10 000 V. Then, the isoelectric focusing continued up to 50 000 Vh. Prior to the 2^nd ^dimension, the focused strip was placed in an equilibration tray with a buffer (6 M urea, 30% (w/v) glycerol, 2% (w/v) SDS, 0.05 M BisTris (pH 6.4)) first with 2% (w/v) DTT for 15 minutes and then with 2.5% (w/v) iodoacetamide at dark for another 15 minutes. The strip was brought on the top of a second dimension resolving gel (10% BisTris gel, 18 cm, 1 mm thickness), overlayered by 4% BisTris stacking gel and subjected to a 2^nd ^dimension at 70 V overnight. Protein spots were visualized by silver staining [25] and the scanned 2-DE gel images sent to the Ludesi Analysis Center (Lund, Sweden, http://www.ludesi.com) for image analysis using proprietary software. Spot detection, segmentation and matching followed a strict protocol to ensure a high level of correctness. The gels were matched using all-to-all spot matching, avoiding introduction of bias caused by the use of a reference gel. The integrated intensity of each of the spot was measured, background corrected and normalized. The normalization removes systematic gel intensity differences originating from variations in staining, scanning time and protein loading by mathematically minimizing the median expression difference between matched spots. Student's t-test was used for calculation of p-values (p < 0.05) for protein spot intensity changes in 2-DE gels. The selection criteria for the candidate spots (spots undergoing subsequent excision, in-gel digestion and LC-MS/MS analysis) were as follows: i) 1.5-fold up (down) spot intensity changes between experimental conditions (e.g. time points within the same incision procedure and between two types of incision procedure but at the same time point), ii) no intensity changes among time points (deviations up to 10% from a mean value), and iii) new spot detected at least at two time points. The candidate spots were excised from 2-DE gels, proteins in spots were trypsin digested and identified by LC-MS/MS.

### In-gel digestion, mass spectrometry and protein identification

For in-gel digestion, the following protocol was applied. The selected protein spots were excised from the silver stained 2-DE gel, cut into about 1 cubic millimeter pieces, destained by using 0.03 M potassium ferricyanide: 0.1 M sodium thiosulfate (1:1) solution, dehydrated with 100% acetonitrile and dried in SpeedVac concentrator to dryness. After destaining, the gel pieces have been further prepared for digestion, first by incubation in reduction agent solution (0.01 M DTT in 0. 25 M ammonium bicarbonate) for 45 minutes at 55°C and then in alkylation solution (0.055 M iodoacetamide in 0.025 M ammonium bicarbonate) for 30 minutes at room temperature. The gel pieces were dehydrated with 100% acetonitrile and dried in SpeedVac concentrator again. Dried gel pieces were covered by trypsin solution (12.5 ng/μL), first by using 20 μl and then additional buffer volume was added in case that the initial volume was absorbed by gel pieces. After incubation for 1 hour at 4°C, the overnight incubation at 37°C took place. After overnight digestion, the excess solution covering the gel pieces was transferred into a vial and resulting tryptic peptides were extracted from the gel pieces by addition of 20 μl of 5% (v/v) formic acid with 15 minutes incubation at room temperature. Then, a volume of 20 μl of 100% acetonitrile was added and incubation was repeated for additional 15 min at room temperature. The whole extraction was repeated twice, and resulting supernatants were pooled and added to the first vial. The pooled extracts were dried to dryness in a vacuum centrifuge. Dried samples with tryptic peptides after trypsin digestion of gel spots were recovered in 9 μl of 0.2% (v/v) trifluoroacetic acid (TFA) and analyzed by a ThermoFinnigan LTQ ion trap with electrospray ionization (Thermo Electron Corporation, MA). The C18 column (120 mm, 75 mm id, YMC ODS-AQ 5 mm particles with 120 A pore size) was used in gradient mode (5-60% of 0.1% formic acid/90% acetonitrile) over 60 min with a flow rate of 300 nL/min. The electrospray voltage was set to 2.3 kV and a data-dependent MS/MS analysis was used. First, an MS survey scan was taken (350-1800 m/z) and the eight most intense parent ions from the survey scan were chosen for consequent MS/MS scans.

Data obtained from MS/MS spectra were submitted to NCBInr database search by using MASCOT search engine (Matrix Science Mascot Daemon, V2.2.0 - max. missed cleavages 2, peptide tolerance ± 1.5 Da and MS/MS tol. ± 0.8 Da, p < 0.05). As the rabbit genome is incomplete and the rabbit protein database is less extensive in number of proteins compared to databases of many other species such as rat, mouse or human, the protein identifications are based as well on sequence homology among species. In our study, we used the protein database search with mammalian taxonomy for protein identifications. After Mascot Daemon search, the files were transferred to Scaffold software (Version Scaffold-01_06_06, 2006 Proteome Software Inc., http://www.proteomesoftware.com, OR) for Mascot result validation, visualization and comparison of protein identifications between individual samples. All identified proteins were further examined for peptide and protein redundancy. The protein amino acid sequence was blasted against UniProt Knowledgebase (Swiss-Prot + TrEMBL) by using SIB BLAST network service (ExPASy). In case of protein multiple names or homology, only one protein name was used after the original peptide sequences obtained from our MS/MS results were checked back for matching that protein by using multiple sequence alignment program ClustalW (EMBL-EBI). Also, the confirmation of a protein isoform was done based on matching a tryptic peptide fragment to a unique amino acid sequence of isoform of the intact protein. In case the same protein name was identified for different species, the peptide sequences were checked and multiply protein name was included only when peptide sequence(s) was unique to species. Positive protein identification was based on at least 2 unique matched peptides or 1 unique matched peptide found in multiple spots.

## Results

Regardless of the method used to determine total protein concentration as shown in Figure 1, there is a large and rapid influx of proteins (within 0.5 hour) into AH after both types of incision with a return to baseline after 12 hours for clear corneal incision and after 24 hours for limbal incision. The limbal incision resulted in a greater increase in protein concentrations compared to clear corneal incision at all time points with highest concentration differences of about 3.2-fold, 2.7-fold and 9.4-fold at 0.5, 2 and 12 hour time points, respectively.

Additional file 1, Figure S1 shows representative 2-DE gel images for AH samples taken after clear corneal incision (panel A) and after limbal incision (Additional file 2, Figure S1, panel B) at 5 time points with pre-surgery AH sample (0 hour). Left column represents low protein amount loaded into gels for individual time points (decrease concentration; 50-78 μg of total protein) and right column shows gels with high protein amount loaded (increase concentration; 280-376 μg of total protein) for corresponding time points with enlargements showing areas exhibiting highest concentration of protein spots. Unfortunately, the same experiments could not be performed at high protein load for AH samples with low protein concentration (pre-surgery samples, and samples at time points 12, 24 and 48 hours for clear corneal incision (Additional file 1, Figure S1, panel A) and samples at time points 24 and 48 hours for limbal incision (Additional file 2, Figure S1, panel B)), due to a limitation in a loading volume for 1^st ^dimension of 2-DE (maximum of 350 μL for 18 cm long strip, see section Methods for more details) and due to a limited amount of AH sample available (approx. 0.2 mL per rabbit). Although the obvious solution of the former problem seems to be the use of concentrating, buffer exchange or centrifugal filtration, we finally opted to use originally collected samples to keep and treat all AH samples the same way. For example, AH contains other components in addition to proteins and using the buffer exchange method would change AH original composition. As well, the spot quantity comparisons between samples would be more complicated if concentration method is used, and using the centrifugal filtration may cause undesirable protein loss.

Additional file 3, Figure S2 shows representative 2-DE gel images with high protein loads of AH samples (280-376 μg of total protein) obtained from clear corneal incision (panel A; 0.5 and 2 hour time points) and limbal incision (panel B; 0.5, 2 and 12 hour time points), 2 different animals per time point are shown.

For 1^st ^dimension of 2-DE, the corresponding volume of AH sample was used to ensure that the similar amount of total protein was loaded into the gels at low concentration (Additional files 1 and 2, Figure S1, panels A and B) or at high concentration (Additional file 3, Figure S2). In order to carry out a quantitative comparison among gels, AH concentration loaded per gel was kept similar, which meant different volumes of the AH were used.

The list of detected proteins is given in Table 1 (the highest number of peptides in spot for corresponding protein are listed if found in multiple gel spot(s)). For proteins detected by 1 peptide, peptide amino sequence and peptide charge are included in Table 1 and they are included only if identified in multiple gel spots, meaning either present in i) different gel spots of appropriate surgery type (clear corneal and/or limbal incision samples), ii) identical spots over different time points within the same type of incision, iii) identical spots but for different type of incision (clear corneal *vs *limbal), and iv) identical spots within the same type of incision but for different protein loads (low *vs *high loads).

**Table 1:** List of unique proteins identified in AH surgery samples.

Protein name	Accession #	Protein type^a^	Surgery type^b^	**Found in **[19]**?**	# of peptides^c^	Peptide sequence^d^	Peptide charge^d^	PIP [%]^e^
Serum albumin [O. cuniculus]^1,2,3,4^	gi|126723746	S	C, L	yes	27			100
Serum albumin [B. taurus]^1^	gi|1351907	S	C, L	yes	3			100
Albumin [M. mulatta]^1^	gi|109074537	S	C, L	yes	2			100
ALB protein [H. sapiens]	gi|25058739	S	L	yes	3			100
SLAM family member 9 [H. sapiens]^1,2,3,4^	gi|74760694	C	C, L	no	1	LATVVPEK	2	95
Apolipoprotein A-I [O. cuniculus]^1,2^	gi|83628258	S	C, L	yes	14			100
Alpha-1-antiproteinase F [O. cuniculus]^1,2^	gi|126722912	S	C, L	yes	7			100
Serum Transferrin Chain A [O. cuniculus]^1,2,3^	gi|15825992	S	C, L	yes	16			100
Tudor domain-containing protein 12 [M. musculus]^1,2,3^	gi|162416223	C	C, L	no	1	SPLSADLKK	2	95
Iodotyrosine dehalogenase 1 protein [M. musculus]^1,2^	gi|21312562	C	C, L	no	1	DATVPDLK	2	94
Histidine-rich glycoprotein [O. cuniculus]^1^	gi|2494026	S	C	yes	3			100
Pancortin 1 [P. troglodytes]^1^	gi|55632585	C	C, L	no	1	DASLLSPR	2	94
Try10-like trypsinogen [M. musculus]^1,2^	gi|51092303	S	C	yes	1	TLDNDIMLIK	2	95
Paraoxonase [O. cuniculus]^1,2^	gi|126722853	S	C, L	no	1	NPPASEVLR	2	95
Mitogen-activated protein kinase 4 [R. norvegicus]^1^	gi|157819181	C	C, L	no	1	SHSFSDPSPK	2	94
Mucin 16 [H. sapiens]^1^	gi|34501467	C	C	no	1	LSTSPIK	2	
DEAD (Asp-Glu-Ala-Asp) box polypeptide 55 [M. musculus]^1^	gi|109734461	C	C	no	1	AMALADR	2	95
Ribosomal protein L24 [M. musculus]^1,3^	gi|94390118	C	C, L	no	1	AAPKQKIVK	1	95
Alpha-2-HS-glycoprotein [O. cuniculus]^1,2^	gi|12644357	S	C, L	yes	3			100
Gamma-fibrinogen chain fragment [H. sapiens]	gi|577055	S	C, L	yes	2			100
Programmed cell death 8 [M. mulatta]^1^	gi|109132217	C	C	no	1	LLIKLKDGR	2	94
FERM [C. familiaris]^1,3^	gi|73994333	C	C, L	no	1	ALTADLPR	2	95
tRNA wybutosine-synthesizing protein 2 [R. norvegicus]^1^	gi|143679922	C	C	no	1	VAVVAEPR	2	95
Adenylate cyclase 6 [M. musculus]^1^	gi|148672233	C	C, L	yes	1	GKEEKAMLAK	1	94
Actin, cytoplasmic 1 (Beta-actin) [O. cuniculus]^1^	gi|231506	C	C, L	yes	2			100
HIST2H3C protein [P. troglodytes]	gi|55626038	C	L	yes	6			100
Vitamin D-binding protein [O. cuniculus]^1^	gi|603499	C	C, L	yes	2			100
Formin-1 (Limb deformity protein) [M. musculus]^1^	gi|158518557	C	C, L	no	1	IIKLLDGKR	2	93
Immunoglobulin kappa chain [O. cuniculus]^1^	gi|1100745	S	L	no	1	VTQGTTSVVQSFNR	2	95
Cytochrome P450, family 2, polypeptide 66 [R. norvegicus]^1,2^	gi|109463861	C	L	no	1	CLVDELR	2	92
E2F transcription factor 4 [M. mulatta]^1,4^	gi|109128874	C	L	no	1	LAADTLAVR	2	92
Protein arginine N-methyltransferase 5 [M. musculus]^1^	gi|32171623	C	C, L	no	1	GITLSVRP	1	93
Alpha-1-antiproteinase E [O. cuniculus]	gi|126722876	S	L	yes	4			100
Preproalbumin [C. porcellus]^1,2^	gi|33518896	S	L	no	2			100
Tubulin beta-2A chain [R. norvegicus]	gi|116242815	C	L	no	6			100
The Structure Of Collagen Type I Chain A [R. norvegicus]	gi|109156929	C	L	no	2			100
Eukaryotic translation elongation factor 1 [M. mulatta]	gi|109071712	C	L	no	2			100
Desmoplakin [B. taurus]	gi|76651410	C	L	yes	3			100
Desmoplakin [R. norvegicus]	gi|10950477841	C	C	yes	2			99
Stratifin [M. mulatta]	gi|108999776	C	L	no	3			100
Gelsolin [M. mulatta]	gi|109110365	C	L	no	3			100
Transthyretin [O. cuniculus]^2^	gi|136466	S	C, L	yes	2			100
Junction plakoglobin [M. mulatta]	gi|109115422	C	C, L	yes	2			100
Hemopexin [O. cuniculus]	gi|1070649	S	C, L	yes	2			99
Complement C3 [C. potcellus]	gi|544053	S	L	yes	2			
Desmoglein 4 [M. musculus]	gi|148664532	C	C, L	yes	2			
Dermcidin preproprotein [M. mulatta]	gi|109096991	S	L	yes	2			100
Cytosolic sialic acid 9-O-acetylesterase [C. familiaris]	gi|73954581	C	C, L	no	1	MELLADK	2	95
SK2 [C. familiaris]^1^	gi|73970454	C	C, L	no	1	IVTVETK	2	90
Platelet-activating factor acetylhydrolase [C. porcellus]	gi|2497686	S	C, L	no	1	WNSPLK	2	93
Filaggrin 2 [H. sapiens]^1^	gi|74755309	C	C, L	no	1	FSNSSSSNEFSK	2	94
Shank-interacting protein-like 1 [C. familiaris]^1^	gi|73974864	C	C, L	no	1	WAALVR	2	94
Collagen, Alpha 3 type VI [C. familiaris]^1^	gi|73990557	C	C, L	no	1	GVSGDRGSK	1	88
Valosin-containing protein [H. sapiens]^1^	gi|11095436	C	C	no	1	GDIFLVR	1	83
Plasminogen [H. sapiens]^1^	gi|38051823	S	C, L	yes	1	EAQLPVIENK	2	91
Sterol O-acyltransferase 1 [R. norvegicus]^1^	gi|13592087	C	C	no	1	QRCPLK	2	93
Peroxisomal membrane protein 2 [B. taurus]^1^	gi|114050981	C	C	no	1	APAASKLR	2	95
Isocitrate dehydrogenase 3 (NAD+) gamma [H. sapiens]^1^	gi|12804901	C	C	no	1	VATVAGSAAK	2	88
HEJ1 [H. sapiens]^1^	gi|14719299	C	C, L	no	1	EQYSAVIIAK	2	94
Olfactory receptor Olr1474 [R. norvegicus]^1^	gi|47575923	C	C	no	1	DMKDALIR	2	95
Bloom syndrome protein [M. mulatta]^1^	gi|109082375	C	C, L	no	1	LFKKLILDK	1	88
Methylmalonyl-CoA mutase [B. taurus]^1^	gi|11544644	C	L	no	1	KVKSSR	1	85
Cationic trypsin-3 [R. norvegicus]^1^	gi|136417	S	C, L	no	2			89
Limbin [C. familiaris]^1^	gi|73951860	C	L	no	1	LASYLSR	2	89
AMMECR1 [H. sapiens]	gi|6063689	?	L	no	1	MAAGCCGVKK	2	85
Olfactory receptor, family 2, member 35 [H. sapiens]^1^	gi|49226830	C	C, L	no	1	VATVIRKG	2	86
DmX-like protein 2 (Rabconnectin-3) [M. musculus]^1^	gi|90109865	C	L	no	1	HTKASSKQPLR	2	88
Aristaless 3 [C. familiaris]^1^	gi|73959917	C	L	no	1	AWGPACGPKLPR	2	82
Lamin B receptor variant [H. sapiens]	gi|62088608	C	C, L	no	1	KMPSRK	2	91
Zipper CG15792-PA [M. mulatta]	gi|109071904	C	C, L	no	1	AESGNPSIQQKIR	2	87
Kallikrein 24 [M. musculus]	gi|8393675	S	C, L	no	1	DKSNDLMLLR	2	87
GTP-binding protein Mx2 [C. familiaris]	gi|7271909	C	C	no	1	LIEGEEIVKK	2	90
Alpha-1-acid glycoprotein [O. cuniculus]	gi|112880	S	L	yes	1	NSVADLLLLR	2	94
Lumican [O. cuniculus]	gi|21542114	S	L	no	1	FNGLQYLR	2	94
Annexin A1 [O. cuniculus]	gi|1703316	C	L	no	1	TPAQFDADELR	2	94
Complement component 8, gamma [O. cuniculus]	gi|126722836	S	L	no	1	YGATGVPGR	2	91
Serotransferrin [O. cuniculus]	gi|6175087	S	C	no	1	VPSXAVVAR	2	92
Crystallin, lambda 1 [O. cuniculus]	gi|126723698	C	C	no	1	QITGALENIR	2	94
Alpha-2-plasmin inhibitor [O. cuniculus]	gi|130488651	S	C	no	1	SKFDPSLTQR	2	96
Ig gamma heavy chain constant region [O. cuniculus]	gi|2136983	S	L	no	1	VYTMGPPR	2	90

^a^Protein type: S - serum protein, C - cellular protein, ? - not determined (as found in protein databases)

^b^Surgery type: C - clear corneal incision, L - limbal incision

^c^Highest number of different peptides in gel spot(s) for corresponding protein if found in multiple gel spots

^d^Peptide sequence and peptide charge are given for 1 peptide if identified in multiple gel spots of clear corneal and/or limbal surgery. As well, 1 peptide hits were multiple observed (multiple MS spectra in one spot) in several individual spots.

^e^Protein identification probability (PIP) as given by Scaffold software; if protein was identified in multiple spots only highest protein probability is listed in Table 1.

^1^identified in multiple gel spots within the same surgery type (clear corneal or limbal incision)

^2^identified in the same spot during different time points within the same type of surgery

^3^identified in the same spot of different type of surgery (clear corneal *vs *limbal incision)

^4^identified in the same spot of the same type of surgery in both low and high protein load (low *vs *high)

**Figure 2:**
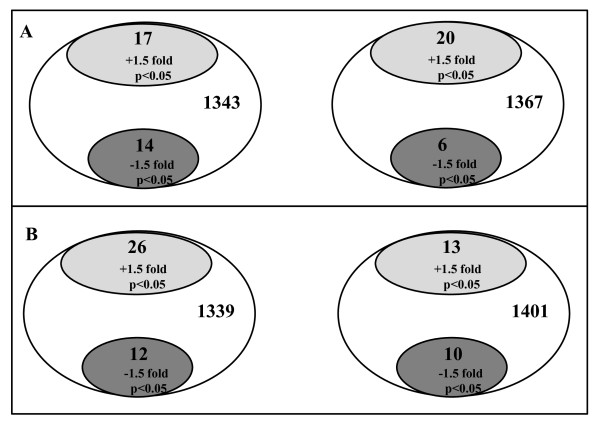
**Number of protein spot changes**. A - from 0.5 to 2 hour time points within clear corneal (left diagram) and limbal (right diagram) incision procedure; B - between clear corneal and limbal incision procedures at 0.5 hour (left diagram) and 2 hour (right diagram) time points. Numbers in white ovals show total protein spots detected by gel image analysis and numbers in grey ovals show number of protein spots which matched our criteria (fold change > ± 1.5 and p-value < 0.05). The p-values were calculated by using Student's t-test.

Based on 2-DE gel image analysis results (Additional file 3, Figure S2), the protein spots that were up- or down-regulated were calculated and Figure 2 shows the examples of the numbers for protein spot changes from 0.5 to 2 hour time points within clear corneal and limbal incision procedures (panel A; fold change > ± 1.5 and p < 0.05; 2 rabbits per time point) and between clear corneal and limbal incisions at 0.5 and 2 hour time points (panel B; fold change > ± 1.5 and p < 0.05; 2 rabbits per time point). As can be seen, from total 1343 protein spots detected for protein spot changes from 0.5 to 2 hour time points within clear corneal incision (panel A, left Venn diagram), only 17 and 14 protein spots, respectively, matched our criteria for up- and down-regulation, and the numbers are similar in magnitude for all four comparisons presented in Figure 2. The examples of proteins identified in several protein spots with fold changes and p-values are listed in Tables 2 and 3 for protein spot changes from 0.5 to 2 hour time point within clear corneal and limbal incision procedures and for protein spot changes between clear corneal and limbal incisions at 0.5 and 2 hour time points, respectively. Proteins included in Table 1 (but not listed in Tables 2 an 3) have been identified either at time points different from 0.5 and 2 hours or in low concentration 2-DE gels (see Additional files 1 and 2, Figure S1, panels A and B). Additional file 4, Figure S3 visualizes the protein spots which changed from 0.5 to 2 hour time point within clear corneal (panel A) and limbal incision (panel B) procedures and between clear corneal and limbal incisions at 2 hour time point (panel C) as detected in 2-DE gels. The proteins identified in individual 2-DE spots can be found in Tables 2 and 3. Additional file 4, Figure S3 shows the zoomed areas for each gel spot to allow easier comparison between corresponding time points and types of incision.

**Table 2:** Protein spot changes from 0.5 hour to 2.0 hour time points within clear corneal (A) and limbal (B) incision procedures.

	spot # (observed pI/M_w _[kDa])	fold change	p-value	proteins identified (accession #; species; theoretical pI/M_w _[Da]; PIP [%])	
				
				at 0.5 hour	at 2.0 hours
**A**	42078 (5.3/44)	-2.2	0.0068	SLAM family member 9 (gi|74760694; H. sapiens; 8.17/30379; 95%)	SLAM family member 9 (gi|74760694; H. sapiens; 8.17/30379; 94%)
				Serum albumin (gi|126723746; O. cuniculus; 5.85/68910; 94%)	
				Albumin (gi|109074537; M. mulatta; 5.91/68874; 95%)	
	
	42147 (6.0/36)	+6.9	0.0040	*	SLAM family member 9 (gi|74760694; H. sapiens; 8.17/30379; 93%)
					Iodotyrosine dehalogenase 1 protein (gi|21312562; M. musculus; 5.97/32814; 89%)
					Mitogen-activated protein kinase 4 (gi|157819181; R. norvegicus; 7.43/35056; 92%)
	
	42151 (4.8/50)	+4.5	0.0004	Try10-like trypsinogen (gi|51092303; M. musculus; 4.83/26531; 95%)	SLAM family member 9 (gi|74760694; H. sapiens; 8.17/30379; 94%)
				Collagen, Alpha 3 type VI (gi|73990557; C. familiaris; 5.43/179948; 88%)	Valosin-containing protein (gi|11095436; H. sapiens; 6.08/34392; 83%)
					Alpha-2-plasmin inhibitor (gi|130488651; O. cuniculus; 5.89/54719; 89%)
	
	42629 (5.1/54)	+2.8	0.0328	Alpha-1-antiproteinase F (gi|126722912; O. cuniculus; 5.83/45868; 100%)	Alpha-1-antiproteinase F (gi|126722912; O. cuniculus; 5.83/45868; 100%)
					SLAM family member 9 (gi|74760694; H. sapiens; 8.17/30379; 95%)
					SK2 (gi|73970454; C. familiaris; 9.78/26354; 83%)
					Olfactory receptor, family 2, member 35 (gi|49226830; H. sapiens; 9.03/36101; 85%)
	
	42696 (5.7/44)	+956	0.0318	**	SLAM family member 9 (gi|74760694; H. sapiens; 8.17/30379; 94%)
					Serum albumin (gi|126723746; O. cuniculus; 5.85/68910; 94%)
					Tudor domain-containing protein 12 (gi|162416223; M. musculus; 6.11/137627; 84%)
					Paraoxonase (gi|126722853; O. cuniculus; 5.51/40010; 94%)

**B**	42064 (5.0/14)	+2.8	0.0039	*	SLAM family member 9 (gi|74760694; H. sapiens; 8.17/30379; 95%)
					Bloom syndrome protein (gi|109082375; M. mulatta; 8.01/158903; 88%)
	
	42092 (4.1/55)	+3957	0.0450	**	SLAM family member 9 (gi|74760694; H. sapiens; 8.17/30379; 90%)
					Iodotyrosine dehalogenase 1 protein (gi|21312562; M. musculus; 5.97/32814; 84%)
					Alpha -2-HS-glycoprotein (gi|12644357; O. cuniculus; 4.99/36696; 95%)
					Plasminogen (gi|38051823; H. sapiens; 6.89/90585; 91%)
					Cytosolic sialic acid 9-O-acetylesterase (gi|73954581; C. familiaris; 6.27/57141; 83%)
	
	42605 (6.5/65)	+7.0	0.0371	Serum albumin (gi|126723746; O. cuniculus; 5.85/68910; 100%)	Serum albumin^a^ (gi|126723746; O. cuniculus; 5.85/68910; 100%)
					SLAM family member 9^a^ (gi|74760694; H. sapiens; 817/30379; 89%)
					Serum Transferrin Chain A^a^ (gi|15825992; O. cuniculus; 6.35/74790; 100%)
					Tudor domain-containing protein 12^a^ (gi|162416223; M. musculus; 6.11/137627; 89%)
					Shank-interacting protein-like 1^a^ (gi|73974864; C. familiaris; 10.39/73215; 89%)
	
	42933 (6.3/29)	+3.5	0.0356	*	SLAM family member 9 (gi|74760694; H. sapiens; 8.17/30379; 95%)
					Olfactory receptor Olr1474 (gi|47575923; R. norvegicus; 8.01/35443; 95%)

For each protein the accession number, species, theoretical pI and M_w _[Da; average] and protein identification probability [%] are given in parenthesis (two last columns of the table). The theoretical pI and M_w _were computed by using Expasy Proteomics server (Swiss Institute of Bioinformatics; http://ca.expasy.org/tools/pi_tool.html).

For each spot the pI and M_w _[kDa] as observed in 2-DE gels are given in parenthesis (2^nd ^column of the table).

*very faint protein spot in 2-DE gel - no proteins identified

**no spot detected by gel image analysis

^a^proteins identified at 12 hour time point

**Table 3:** Protein spot changes between clear corneal and limbal incisions at 0.5 hour (A) and 2 hour (B) time points.

	spot # (observed pI/M_w _[kDa])	fold change	p-value	proteins identified (accession #; species; theoretical pI/M_w _[Da]; PIP [%])	
				
				clear corneal incision	limbal incision
**A**	42558 (6.2/60)	-1.6	0.0219	SLAM family member 9 (gi|74760694; H. sapiens; 8.17/30379; 95%)	*
				Serum albumin (gi|126723746; O. cuniculus; 5.85/68910; 100%)	
				Mucin 16 (gi|34501467; H. sapiens; 5.51/744966; 85%)	

**B**	42543 (5.6/18)	+2.8	0.0366	SLAM family member 9 (gi|74760694; H. sapiens; 8.17/30379; 95%)	SLAM family member 9 (gi|74760694; H. sapiens; 8.17/30379; 93%)
				FERM (gi|73994333; C. familiaris; 9.13/117880; 95%)	Serum albumin (gi|126723746; O. cuniculus; 5.85/68910; 100%)
				GTP-binding protein Mx2 (gi|7271909; C. familiaris; 8.33/81442; 90%)	E2F transcription factor 4 (gi|109128874; M. mulatta; 4.66/44295; 92%)
	
	42727 (5.5/18)	-2.2	0.0012	SLAM family member 9 (gi|74760694; H. sapiens; 8.17/30379; 95%)	*
				Iodotyrosine dehalogenase 1 protein (gi|21312562; M. musculus; 5.97/32814; 88%)	
	
	43222 (4.2/65)	+2697	0.0095	**	SLAM family member 9 (gi|74760694; H. sapiens; 817/30379; 89%)
					Serum albumin (gi|126723746; O. cuniculus; 5.85/68910; 100%)
					HIST2H3C protein (gi|55626038; P. troglodytes; 10.50/60795; 100%)
					Desmoglein 4 (gi|148664532; M. musculus; 4.61/102815; 100%)

For each protein the accession number, species, theoretical pI and M_w _[Da; average] and protein identification probability [%] are given in parenthesis (two last columns of the table). The theoretical pI and M_w _were computed by using Expasy Proteomics server (Swiss Institute of Bioinformatics; http://ca.expasy.org/tools/pi_tool.html).

For each spot the pI and M_w _[kDa] as observed in 2-DE gels is given in parenthesis (2^nd ^column of the table).

*very faint spot in 2-DE gel - no proteins identified

**no spot detected by gel image analysis

**Figure 3:**
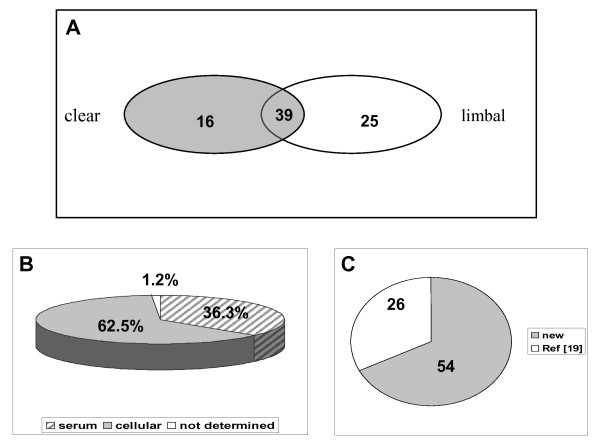
**Distribution of 80 unique proteins**. A - proteins identified in clear corneal and limbal incision AH samples; B - cellular, serum and origin not determined proteins; C - proteins already identified in healthy AH (previous Ref [19]) and proteins newly found in this study.

Figure 3 shows the distribution of 80 identified unique proteins. In panel A, 16 and 25 proteins were identified in clear corneal and limbal incision samples, respectively, and 39 proteins were common to both types of surgery (see Table 1, 4^th ^column). Protein database search and literature search revealed that 62.5% and 36.3% were cellular and serum proteins, respectively, location was not determined for 1.2% of proteins (Figure 3, panel B and Table 1, 3^rd ^column). Compared to our previous study (Ref. [19]), in which we listed the proteins detected in healthy AH of rabbits, only 26 proteins have been common to both our studies, whereas 54 proteins (67.5%) were newly detected here in surgery AH samples (Figure 3, panel C and Table 1, 5^th ^column).

Proteins found in AH surgery samples and specific only for one type surgery (either clear corneal or limbal incision) but not identified in healthy AH (Ref [19]) were: mucin 16, DEAD box polypeptide 55, programmed cell death 8, tRNA wybutosine-synthesizing protein 2, cytochrome P450, E2F transcription factor 4, tubulin, collagen type I, eukaryotic translation elongation factor 1, stratifin, gelsolin, valosin-containing protein, sterol O-acyltransferase 1, peroxisomal membrane protein 2, isocitrate dehydrogenase 3, olfactory receptor Olr1474, methylmalonyl-CoA mutase, limbin, AMMECR1, rabconnectin-3, aristaless 3, lumican, annexin A1, complement component 8 and crystallin lambda 1. Majority of these proteins are cellular proteins, (88%; based on Protein Knowledgebase (UniProtKB/Swiss-Prot), Gene Ontology database and literature search), only two of them are serum proteins (lumican and complement component 8). For example, 5 cellular proteins, i.e. tubulin, collagen type I, eukaryotic translation elongation factor 1, stratifin (also called 14-3-3 protein sigma) and gelsolin, were exclusively present in AH samples undergoing limbal incision.

Proteins identified in the identical protein spots of samples collected over various time points (corresponding protein spots are incised from different time point gels) were either serum proteins - serum albumin, apolipoprotein A-I, alpha-1-antiproteinase F, transferrin, Try 10-like trypsinogen, paraoxonase, alpha-2-HS-glycoprotein, transhyretin or cellular proteins - SLAM family member 9, tudor domain-containing protein 12, iodotyrosine dehalogenase 1 protein, cytochrome P450, and peroxisomal membrane protein 2. The proteins were present in AH during various period of time after surgery, e.g. SLAM family member 9 was found in samples collected at 0.5, 2 and 12 hours after both clear corneal and limbal incision, tudor domain-containing protein 12 and cytochrome P450 in samples after limbal incision at time points 0.5, 2, 12 and 24 hours and 12 and 24 hours, respectively, and paraoxonase and peroxisomal membrane protein 2 were present in samples collected at 0.5 and 2 hours following clear corneal incisison.

To obtain more extensive proteome coverage, 2-DE with high protein load were performed for AH samples collected at time points 0.5-2 hours (clear corneal incision) and 0.5-12 hours (limbal incision), respectively (Additional file 3: Figure S2). We identified additional 14 proteins (not identified in healthy AH samples) which were only detected with high but not with low protein load.

## Discussion

After surgery, a large concentration of proteins entered the AH in a relatively short time (0.5 hour). This does not appear to be a selection based upon protein size or charge, but rather a flood of proteins entering AH. For example, we identified proteins ranging from molecular weight 32814 Da (iodotyrosine dehalogenase 1 protein) to 248073 Da (fillagrin 2; Protein Knowledgebase (UniProtKB/Swiss-Prot)). As well, it corresponds to the 2-DE gels showing protein spots spread across relatively wide Mw (6 to 200 kDa) and pI ranges (4 to 7; Additional files 1, 2 and 3, Figures S1 and S2).

Figure 2 shows that the numbers of up- and down- regulated protein spots from total spot detected are in similar magnitude for all four comparisons presented. Only a few protein spots from over 1000 total protein spots were found to be changed (from 6 to 26 protein spots, depending on comparison) between the time points within the same incision procedure and between two surgery procedures evaluated at the same time points. However, the limitation of the study is that only a subset of these altered protein spots was successfully identified although many attempts were made including the combination the same spots from number of gels and use of different MS instruments. This may be due to the facts that i) most spots contained more than one protein and ii) that many of the spots that changed were different between compared groups (either the time points within the incision procedure or between two surgery procedures at the same time point; for example see Tables 2 and 3). Thus, it was not possible to unambiguously assign quantitative changes to one protein in the vast majority of the cases. Rather it can be only stated that group of proteins was found in protein spot which was either up- or down-regulated.

The differences between protein theoretical pI and M_w _values and values observed by 2-DE gels for each corresponding protein spot (Tables 2 and 3) can be explained by a number of different parameters. First, by species differences, since the species other from rabbit were identified for many proteins and amino acid sequence differences can change both pI and M_w_. Second, the PTM can also shift pI and Mw. The observed M_w _values compared to theoretical M_w _values were lower for many proteins, which suggests that protein degradation (proteolysis) was involved. For example, tudor domain- containing protein 12 and serum albumin with theoretical M_w _of 137627 Da and 68910 Da were detected in 2-DE protein spots with M_w _of 44 kDa and 65 kDa (Table 2) and M_w _of 18 kDa (Table 3), respectively. On the contrary, SLAM family member 9 with theoretical M_w _value of 30379 Da was identified in many 2-DE protein spots of higher M_w _(e.g. 44, 50, 55 and 65 kDa (Table 2)) which can be accounted for the fact that SLAM family member 9 occurs in two isoforms varying by approximately 10 kDa in M_w _in human (22.6 kDa and 32.4 kDa) and that it is a glycoprotein with 2 to 6 modifiable amino acid sites in human and mouse species, respectively (Protein Knowledgebase (UniProtKB/Swiss-Prot)).

Of the total 46 proteins identified in AH samples collected at 0.5 hour after both types of incisions, 20 proteins (44%) and 13 proteins (28%) were clear corneal and limbal incision specific, respectively, with only 13 proteins found in both types of incisions.

From a total of 45 proteins identified in AH samples collected 2 hours after both types of surgeries, 22 (49%) and 10 (22%) proteins were clear corneal and limbal incision specific, respectively, with 13 proteins common to both surgery types. Based on these results, there is not a straightforward answer to the question of whether differences in AH protein concentrations between both types of surgeries (at least for AH samples from 0.5 and 2 hours) are due to a different set and numbers of proteins in each type of surgery or due to same proteins but of various concentrations or due to the combination of both cases. Since the protein concentrations are much higher in AH samples undergoing limbal incision compared to AH samples after clear corneal incision (compared 31.2 mg/mL and 21.4 mg/mL to 9.7 mg/mL and 8.0 mg/mL for 0.5 and 2 hours, respectively; Figure 1, Bradford Bio-Rad protein assay), and the number of proteins specific for each type of surgery is quite distinct (i.e. 13 and 10 proteins specific for limbal incision and 20 and 22 proteins specific for clear corneal incision for 0.5 and 2 hour samples), the higher concentrations of the proteins rather than number of different proteins probably contribute to the protein content, at least for AH samples after limbal incision.

Although we identified proteins from the serum, the majority are of cellular origin (Table 1). Unlike the protein composition of healthy AH which arises from specific cell types and could, at least a subset, be assigned to protective functions (36%) [19], cellular proteins found in AH after surgery could not be assigned, in most cases, to any particular cell or function (based on protein databases and literature search) except for a number of proteins including mucin 16, SLAM family member 9, annexin A1 and crystalline lambda 1. Mucin 16 is thought to provide a protective, lubricating barrier against particles and infectious agents at mucosal surfaces and it is expressed in corneal and conjunctival epithelia. SLAM family member 9 may play a role in immune response and it is expressed and found in membrane of immune cells. Annexin A1 is expressed in ciliated cells in lung tracheal endothelium and crystalline lambda 1 is a major rabbit lens protein. None of these proteins were identified in healthy AH [19] and, except for SLAM family member 9, neither in pre-surgery samples. Clear corneal incision specific proteins mucin 16 and crystalline lambda 1 were still detected in AH samples after 2 hours of surgery as well as limbal incision specific annexin A1. SLAM family member 9, which is not specific for either type of surgery, was present in pre-surgery AH samples and remained present in AH samples even after 24 hours following surgery.

Ten "protective" proteins which have been presented in healthy AH [19] were identified in surgery samples as well. The proteins belong to four functional groups (cell/cell interactions/wound healing, proteases and protease inhibitors, antioxidant protection, and antibacterial/anti-inflammatory proteins) [19]. They are alpha-1-antiproteinase F, transferrin, trypsinogen, desmoplakin, transhyretin, plakoglobin, hemopexin, complement C3, desmoglein and dermcidin. Of those, only 4 proteins were type incision specific: trypsinogen and desmoplakin were found only in samples collected after clear corneal incision, while complement C3 and dermcidin were identified in samples undergoing the limbal incision. While trypsinogen and desmoplakin were found in AH samples after 2 hours of surgery, dermcidin was still present in AH samples collected after 12 hours. Surgery non-specific proteins transhyretin, plakoglobin and desmoglein were indentified in AH samples after 48 hours following the surgery. Nevertheless, the fact that most of the protective proteins detected in healthy AH [19] is not seen in surgery AH samples could imply that a mechanism of protein release into AH after surgery can be rather a global response to the surgery than the increase in amount of protective proteins found in healthy AH as one could expect.

Compared to list of proteins detected in other most recent studies on proteomic analysis of human AH [17,18], only 27 of total 80 proteins in Table 1 were previously identified, but not for rabbit species (O. cuniculus) as in our study.

## Conclusions

We identified 80 unique proteins in samples of rabbit AH collected at various time points following clear corneal and limbal incision procedures during a cataract surgery by 2-DE and LC-MS/MS. 67.5% of these proteins have been found only in AH samples undergoing cataract surgery as compared to our previous protein database on healthy rabbit AH [19]. In addition, 51% of proteins have been found either in clear corneal incision (20%) or limbal incision (31%) which suggests that the mechanism of the protein release for each type of cataract surgery incision procedure could be, at least partially, different. However, only a small number of protein spots changed between the 0.5 and 2 hour time points and between two different surgical methods (0.4 - 1.9%). Although the total protein concentration was increased, many of 2-DE protein spots were similar between conditions and there were only a few unique proteins per time point and surgery type (based on 2-DE and MS analyses). This suggests that the high protein content in AH samples is due to high concentration of the same proteins with only a few unique proteins per time point and surgery type. Most of the proteins that were altered were not known to be involved in protection implying that there is a global influx of proteins into AH rather than increase in amount of protective proteins. As well, the number of surgery specific proteins identified in this study increases the number of protein identifications in AH known up to now.

## Competing interests

The authors declare that they have no competing interests.

## Authors' contributions

MS wrote the main manuscript and designed and performed the most of the experiments. AB planned and participated in AH collection, contributed to the design of the study and revision of the manuscript draft. PJM contributed to the design of the study, data interpretation, and manuscript writing. JEV participated in the design of the experiments, supervised the data analysis and interpretation, and participated in manuscript writing. All authors read and approved the final manuscript.

## Supplementary Material

Additional file 1 **Figure S1, panel A**. Representative 2-DE gel images of AH samples (with low protein loads) collected at five time points after clear corneal incision. The gel images of AH samples with high protein loads for corresponding time points are shown as well for better comparison of protein spot pattern. Gels (x-axis): pI 4-7, (y-axis): M_w _6-200 kDa (as marked by protein markers on the left side of each gel). Detailed explanation in the text. Click here for file

Additional file 2 **Figure S1, panel B**. Representative 2-DE gel images of AH samples (with low protein loads) collected at five time points after limbal incision. The gel images of AH samples with high protein loads for corresponding time points are shown as well for better comparison of protein spot pattern. Gels (x-axis): pI 4-7, (y-axis): M_w _6-200 kDa (as marked by protein markers on the left side of each gel). Detailed explanation in the text. Click here for file

Additional file 3 **Figure S2**. Representative 2-DE gel images of AH samples (with high protein loads) after: A - clear corneal incision (0.5 and 2 hour time points) and B - limbal incision (0.5, 2 and 12 hour time points); each time point for two animals. Gels (x-axis): pI 4-7, (y-axis): M_w _6-200 kDa (as marked by protein markers on the left side of each gel). More details can be found in the text. Click here for file

Additional file 4 **Figure S3**. The visualization of 2-DE protein spots which changed from 0.5 to 2 hour time point within clear corneal (panel A) and limbal incision (panel B) procedures and between clear corneal and limbal incisions at 2 hour time points (panel C); see Table 2 and Table 3 for protein identifications. More details are included in the text. Click here for file
