# Supplementary figures and images for: Analysis of protein composition of rabbit aqueous humor following two different cataract surgery incision procedures using 2-DE and LC-MS/MS
Source: Proteome Sci. 2011 Feb 9;9:8. doi: 10.1186/1477-5956-9-8 (PMC3045281; doi:10.1186/1477-5956-9-8)

Figure S1

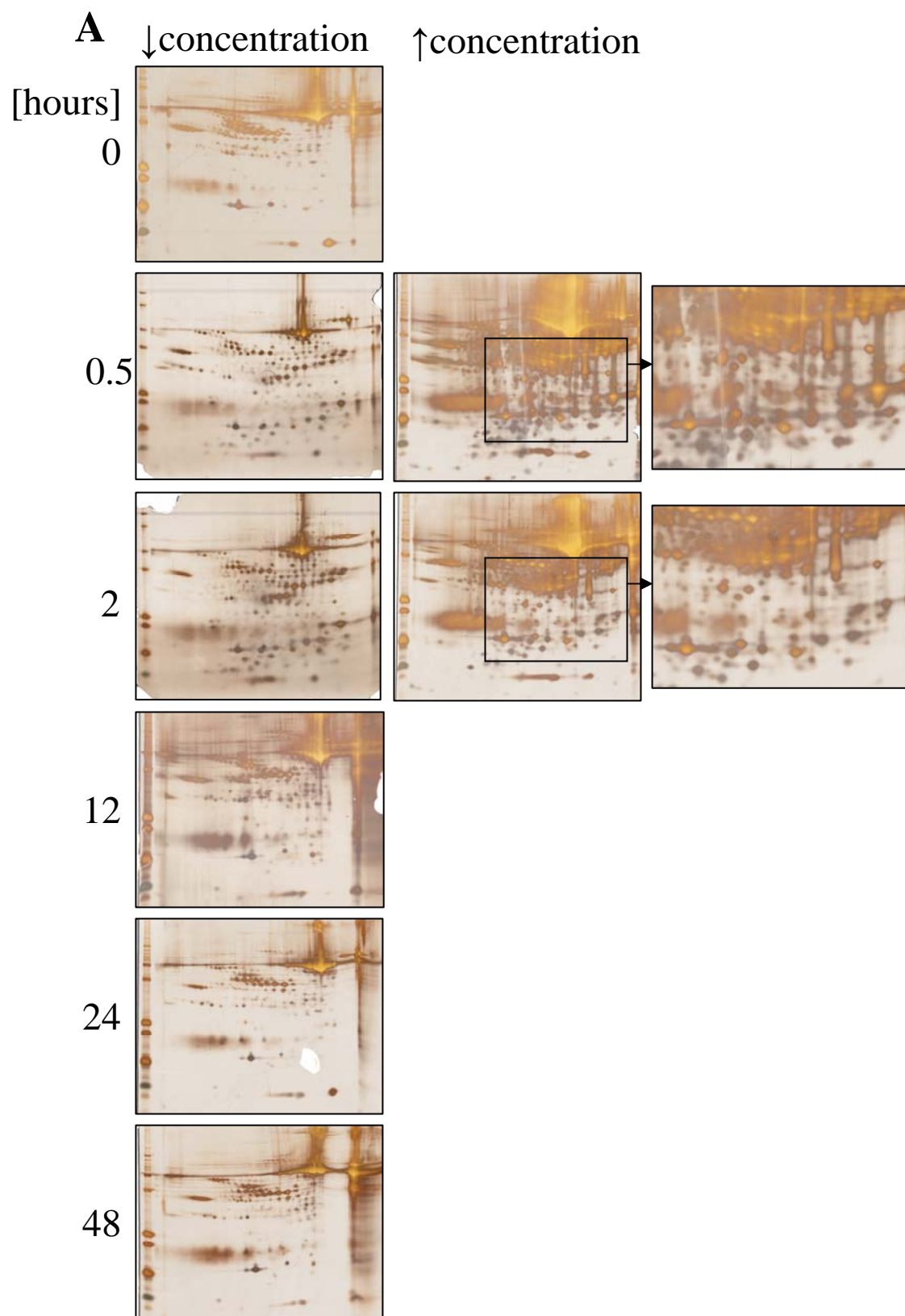

Supplement: Additional file 1 — Figure S1, panel A. Representative 2-DE gel images of AH samples (with low protein loads) collected at five time points after clear corneal incision. The gel images of AH samples with high protein loads for corresponding time points are shown as well for better comparison of protein spot pattern. Gels (x-axis): pI 4-7, (y-axis): Mw 6-200 kDa (as marked by protein markers on the left side of each gel). Detailed explanation in the text. [file 1477-5956-9-8-S1.PDF]

Figure S1

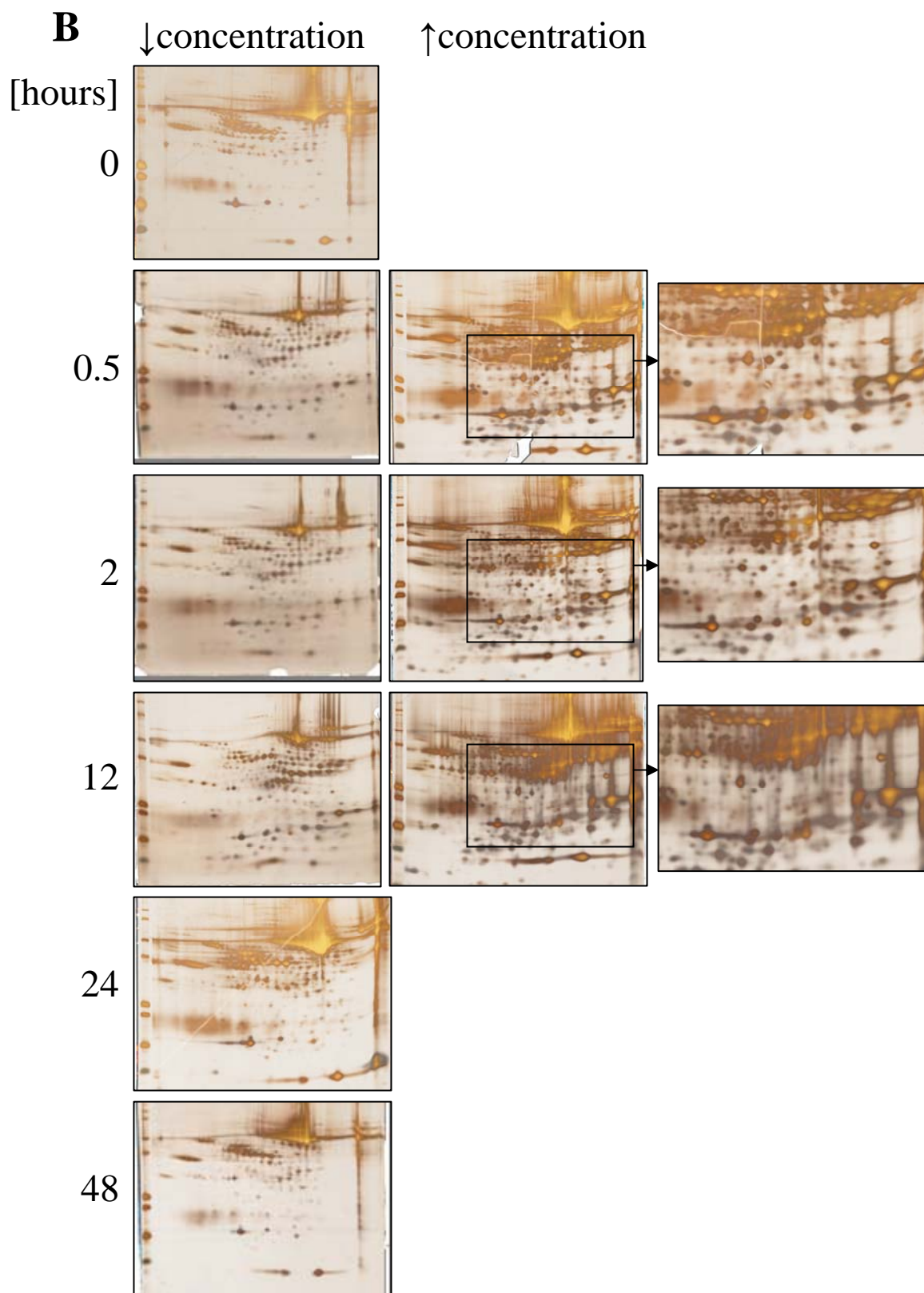

Supplement: Additional file 2 — Figure S1, panel B. Representative 2-DE gel images of AH samples (with low protein loads) collected at five time points after limbal incision. The gel images of AH samples with high protein loads for corresponding time points are shown as well for better comparison of protein spot pattern. Gels (x-axis): pI 4-7, (y-axis): Mw 6-200 kDa (as marked by protein markers on the left side of each gel). Detailed explanation in the text. [file 1477-5956-9-8-S2.PDF]

Figure S2

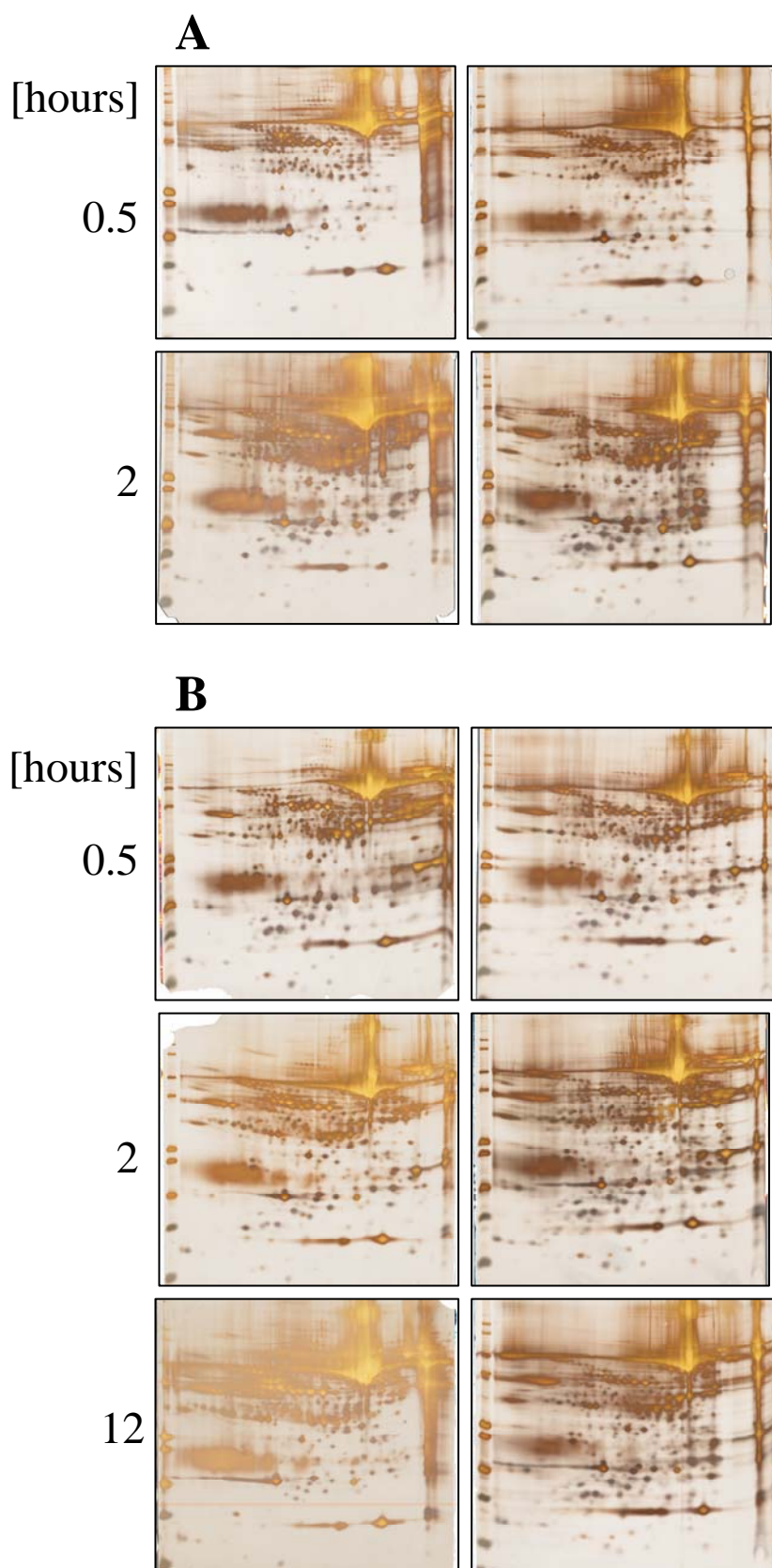

Supplement: Additional file 3 — Figure S2. Representative 2-DE gel images of AH samples (with high protein loads) after: A - clear corneal incision (0.5 and 2 hour time points) and B - limbal incision (0.5, 2 and 12 hour time points); each time point for two animals. Gels (x-axis): pI 4-7, (y-axis): Mw 6-200 kDa (as marked by protein markers on the left side of each gel). More details can be found in the text. [file 1477-5956-9-8-S3.PDF]

Figure S3

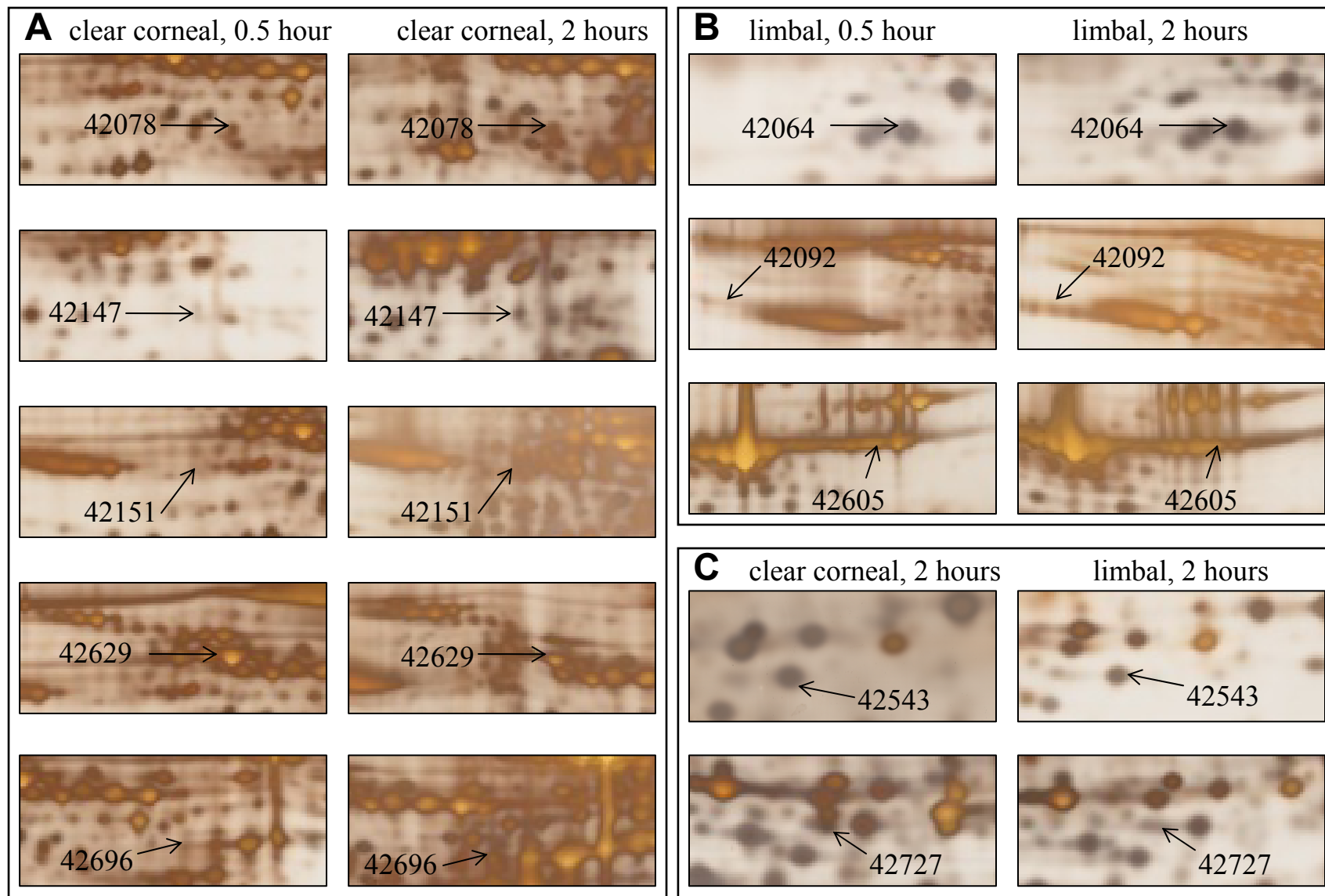

Supplement: Additional file 4 — Figure S3. The visualization of 2-DE protein spots which changed from 0.5 to 2 hour time point within clear corneal (panel A) and limbal incision (panel B) procedures and between clear corneal and limbal incisions at 2 hour time points (panel C); see Table 2 and Table 3 for protein identifications. More details are included in the text. [file 1477-5956-9-8-S4.PDF]
